# Application of the Consolidated Framework for Implementation Research to examine nurses’ perception of the task shifting strategy for hypertension control trial in Ghana

**DOI:** 10.1186/s12913-020-4912-5

**Published:** 2020-01-29

**Authors:** Joyce Gyamfi, John P. Allegrante, Juliet Iwelunmor, Olajide Williams, Jacob Plange-Rhule, Sarah Blackstone, Michael Ntim, Kingsley Apusiga, Emmanuel Peprah, Gbenga Ogedegbe

**Affiliations:** 1 Department of Health and Behavior Studies, Teachers College, Columbia University, 525 West 120 Street, New York, NY 10027 USA; 2 Behavioral Science and Health Education, College for Public Health and Social Justice, Salus Center, Saint Louis University, 3545 Lafayette Ave., Saint Louis, MO 63103 USA; 3 Columbia University Medical Center, 710 West 168th Street, New York, NY 10032 USA; 4 Department of Physiology, Kwame Nkrumah University of Science and Technology, Private Mail Bag University Post Office KNUST, Kumasi, Ghana; 5 Health and Behavioral Studies Building, James Madison University, MSC 4301,235 Martin Luther King Jr Way, Harrisonburg, VA 22807 USA; 6 College of Global Public Health, New York University, 726 Broadway, New York, NY 10012 USA; 7 Department of Population Health/ Section for Global Health, NYU School of Medicine, 180 Madison Avenue, New York, NY 10016 USA

**Keywords:** Hypertension, Task shifting, Blood pressure control, Community health centers, Community health nurses, Ghana, Consolidated framework for implementation research, CFIR, Low-resource settings

## Abstract

**Background:**

The burden of hypertension in many low-and middle-income countries is alarming and requires effective evidence-based preventative strategies that is carefully appraised and accepted by key stakeholders to ensure successful implementation and sustainability. We assessed nurses’ perceptions of a recently completed Task Shifting Strategy for Hypertension control (TASSH) trial in Ghana, and facilitators and challenges to TASSH implementation.

**Methods:**

Focus group sessions and in-depth interviews were conducted with 27 community health nurses from participating health centers and district hospitals involved in the TASSH trial implemented in the Ashanti Region, Ghana, West Africa from 2012 to 2017. TASSH evaluated the comparative effectiveness of the WHO-PEN program versus provision of health insurance for blood pressure reduction in hypertensive adults. Qualitative data were analyzed using open and axial coding techniques with emerging themes mapped onto the Consolidated Framework for Implementation Research (CFIR).

**Results:**

Three themes emerged following deductive analysis using CFIR, including: (1) *Patient health goal setting-* relative priority and positive feedback from nurses, which motivated patients to make healthy behavior changes as a result of their health being a priority; (2) *Leadership engagement* (i.e., medical directors) which influenced the extent to which nurses were able to successfully implement TASSH in their various facilities, with most directors being very supportive; and (3) *Availability of resources* making it possible to implement the TASSH protocol, with limited space and personnel time to carry out TASSH duties, limited blood pressure (BP) monitoring equipment, and transportation, listed as barriers to effective implementation.

**Conclusion:**

Assessing stakeholders’ perception of the TASSH implementation process guided by CFIR is crucial as it provides a platform for the nurses to thoroughly evaluate the task shifting program, while considering the local context in which the program is implemented. The feedback from the nurses informed barriers and facilitators to implementation of TASSH within the current healthcare system, and suggested system level changes needed prior to scale-up of TASSH to other regions in Ghana with potential for long-term sustainment of the task shifting intervention.

**Trial registration:**

Trial registration for parent TASSH study: NCT01802372. Registered February 27, 2013.

## Background

Implementing effective evidence-based health interventions for hypertension control in Sub-Saharan African (SSA) countries like Ghana is urgent considering the alarming increase in hypertension related morbidity and mortality. Approximately 65% of the 972 million people with hypertension live in the developing world, which is estimated to increase to 1.5 billion by the year 2025 [1, 2]. Recent systematic reviews estimate the overall prevalence of hypertension in low- and middle- income countries (LMICs) at 32.3% [3] and 25–41% for SSA [4]. Moreover, by 2020, 75% of all deaths in SSA will be attributed to hypertension [5], and in Ghana, hypertension is the second leading cause of morbidity in adults 45 years and older [6, 7]. The prevalence of hypertension in Ghana ranges from 19.3% in rural areas to 54.6% in urban areas [8]. Thus, there is a pressing need for effective preventative strategies in an effort to control hypertension. Moreover, it is imperative that health interventions are carefully appraised and accepted by key stakeholders, including site directors, patients, and nurses in order to ensure successful implementation and sustainability. Thereby avoiding the pitfall of ‘one shot deal’ programs occurring with interventions which fail to assess the implementation process [9] in LMICs.

However, many LMICs such as Ghana lack the capacity to implement and sustain evidence-based intervention strategies, largely due to shortage of physicians [7]. In SSA, there are 2.4 million doctors and nurses, which translate to 2 doctors and 11 nursing/midwifery personnel per 10,000 people, compared to 19 doctors and 49 nursing/midwifery personnel per 10,000 in North America [10]. Ghana, like most countries in SSA is affected by the “brain-drain,” defined as high rate of emigration among physicians for various reasons including dissatisfaction with working conditions [11]. According to findings from Opoku and colleagues (2014) study in Ghana, physicians who were dissatisfied with available resources, compensation and work-life balance, reported an intent to leave Ghana within 5 years [12], which will further exacerbate the physician shortage. An effective strategy to deal with the shortage of physicians is task shifting of health care duties from physicians to non-physician healthcare providers (NPHCPs) at the primary care level [13]. According to the World Health Organization (WHO), task shifting is a process of delegation whereby tasks are moved, when appropriate, to less specialized health workers [14]. This is a cost effective strategy appropriate for areas lacking healthcare resources and personnel [7]. Task shifting has shown to be effective in the context of HIV management [15, 16], as well as cardiovascular disease [17, 18] in SSA. While there is evidence that task shifting strategies can be successful in aiding disease management in low resource settings, there is little evidence regarding key stakeholders’ perceptions of task shifting implementation. Involving stakeholders, such as health care providers in the research process, is imperative to ensuring successful implementation and continuation of interventions beyond initial implementation [9].

Recently a task shifting strategy for hypertension (TASSH) management trial was completed in Ghana (Trial registration: NCT01802372) [7, 19]. This program aim at mitigating the burden of hypertension in the Kumasi area, the Ashanti region of Ghana. As part of the study protocol, nurses from 32 district hospitals and community health centers (CHCs) randomized to intervention and control groups received training in the World Health Organization Package of Essential Non-communicable Disease Intervention for Primary Care (WHO-PEN). The training provided the nurses practical skills to enable them to diagnose, treat, and manage uncomplicated cases of hypertension that normally would be under the care of physicians [7]. The WHO-PEN is an innovative and action oriented task shifting strategy designed to provide care to patients who live in an environment with a low resource health care system, and high out-of-pocket expenditure for health services such as in Ghana [14, 16]. WHO-PEN is an evidence-based program that provides clinical decision support for assessing and managing cardiovascular risk. The program provides risk assessment algorithms, lifestyle counseling, drug treatment protocols, and referral networks [14]. In evaluating the WHO-PEN program’s effectiveness for managing hypertension in Nigeria and China, Mendis and colleagues found that the program was successful in reducing both systolic and diastolic blood pressure [20]. Although this evaluation showed the positive effect of task shifting on BP management at the patient level, data regarding stakeholders’ perceptions of the intervention implementation and service delivery are lacking. Furthermore, as nurses are the cornerstone of many task shifting programs, including TASSH, understanding their perceptions of program implementation can provide crucial information to improving the uptake of effective health interventions. Thus, the purpose of this study was to identify and describe the community health nurses’(CHNs) perceptions of facilitators and challenges faced with TASSH implementation.

### Conceptual framework

We employed the Consolidated Framework for Implementation Research (CFIR) to explore nurses’ perception of TASSH. CFIR was developed by Damschroder and colleagues in 2009 [9], as it offers an overarching typology to promote theoretical development regarding what is effective and what is not across different implementation settings. Five domains comprise the CFIR: intervention characteristics (e.g., evidence strength and quality, adaptability), outer setting (e.g., patient needs and resources, peer pressure), inner setting (e.g., structural characteristics, networks and communication), characteristics of individuals involved (e.g., stages of change, self-efficacy), and the process of implementation (e.g., planning and executing an intervention protocol). This study focused on two constructs of inner setting - implementation climate and readiness for implementation - to examine TASSH implementation from the perspective of participating CHNs. Implementation climate refers to the extent to which an intervention will be rewarded and supported within an organization [9, 21].

Related to implementation climate is readiness for implementation of an intervention, which was also examined in relation to TASSH. Readiness for implementation is differentiated from implementation climate by tangible indicators of organizational commitment and desire to implement an intervention. It consists of three subconstructs: *leadership engagement*, or the commitment, involvement, and accountability of leaders and managers in implementation [9, 22, 23]; *available resources*, or the level of resources dedicated for implementation (e.g., funds, equipment, personnel, training) [9, 24, 25], and *access to information and knowledge*, or the degree to which information about the intervention implementation is easily accessible and digestible [9], and use of that knowledge as evident through patient health goal setting. The aim of this article was to use the CFIR model to assess nurses’ perceptions of the task shifting strategy for blood pressure control in Ghana, and facilitators and challenges to TASSH implementation. The results will be informative for identifying successes and challenges prior to widespread implementation of TASSH in Ghana.

## Methods

### Participants

Semi-structured, in-depth interviews and focus group sessions were conducted with 27 TASSH nurses attending TASSH training at Kwame Nkrumah University of Science and Technology (KNUST) in Kumasi, Ghana. We used purposive sampling [26] to recruit trained nurses from both the treatment and control sites, working to implement the WHO-PEN program and manage uncomplicated cases of hypertension in 32 community health centers and district hospitals. Details of the TASSH trial is published elsewhere [7], however, in brief, TASSH was a 5-year cluster-randomized trial completed in 2017. The program was implemented in Kumasi, located in the Ashanti Region of Ghana, to facilitate high blood pressure reduction in this population and equip participants with pertinent health behavior knowledge to ensure adequate long-term management of their condition. For the parent TASSH study, 64 TASSH nurses (two from each site, intervention and control) participated in at least five training sessions in which they were trained in the WHO-PEN package [7, 27]. Inclusion criteria for the current qualitative study were that participants must be at least 18 years of age and participated in the TASSH training program. Thirty-two nurses were asked in-person to participate in this study during a final training session, 27 nurses agreed and were consented. Six nurses could not participate as a result of time constraints. The study received ethical approval from the New York University School of Medicine and the Kwame Nkrumah University of Science and Technology Institutional Review Boards. Prior to the interviews, nurses provided written and verbal informed consent after being given a brief overview of the study purposes, and were made aware that participation in the study had no bearing on their current or future work with TASSH and the health facilities.

### Data collection

#### Interviews

Twenty-seven nurses participated in the semi-structured, in-depth interviews. The nurses represented 18 participating health facilities (with at least one nurse each from 13 sites and seven sites providing two nurses each). An interview guide was developed based on the CFIR model focusing on resources needed to implement and administer TASSH, leadership engagement during TASSH, and overall patients’ and nurses’ experiences with the TASSH program. The interview guide was verbally translated from English to Twi, the local dialect of the Ashanti region. The lead author (JG, female), who was the Senior Research Coordinator at the time of data collection and two masters-level bilingual (English-Twi) research coordinators (MN and KA, males) trained in qualitative interviewing conducted the interviews. The facilitators had a keen interest in understanding the holistic experience from the nurses’ viewpoint with the TASSH implementation. Each interview lasted up to 30 min. A semi-structured guide with open-ended questions was used for all discussions. This approach allowed interviewers to tailor questions and probes as needed for the different participants. The open-ended questions also allowed participants to elaborate on issues they considered important or relevant. Interviews were transcribed and translated into English for data analysis.

#### Focus groups

Two focus group sessions (groups of 14 and 13 with an equal mix of representation from the various facilities) were conducted with the same participants who provided responses to the individual interviews. The focus group discussions lasted 60  min. Questions posed to the nurses were: What were the barriers and/or facilitators to TASSH implementation? Can you describe your personal experience with the TASSH program? What information or support did you receive as part of the TASSH program? Was it helpful or unhelpful? Why? Focus group sessions were audiotaped and professionally transcribed verbatim, and transcripts were checked for accuracy. Data were collected from this group until theme saturation. Interviews were transcribed and translated into English for data analysis.

The Consolidated Criteria for Reporting Qualitative Research (COREQ) [28] guided the reporting of the qualitative data. Information on research teams conducting the interviews and focus groups, study design and data analysis and results are provided. Furthermore, this qualitative study “emphasize[s] the voices of participants through quotes” [29].

### Data analysis

Descriptive statistics including mean and or frequencies for age, gender, and education level were summarized for the nurses involved in the in-depth structured interviews and focus group sessions, using SPSS statistical software. The qualitative data were analyzed using an iterative process. The statements from the in-depth interviews were transcribed verbatim and exported into Microsoft Excel. We coded the data using open and axial coding techniques advocated by Corbin and Strauss [30]. During open coding, three researchers read through the interview transcript and extracted key words and phrases related to the research question. Together, the research team compared the key words and phrases across participants and decided on final labels to identify the concepts. Following open coding, axial coding entailed grouping concepts into sub-themes and deciding on final thematic categories to address the research question. Data were coded deductively, using CFIR as a guide during theme generation. Member checking was used during and after data collection to verify the information collected and interpretation of our findings. Member checking involves returning to the participants to ensure that the researchers’ interpretations aligned with participants’ intended messages [31].

## Results

### Site and participant characteristics

A total of 27 CHNs were interviewed from various sites representative of urban and rural facilities from the Ashanti region included in the parent TASSH study (Table 1). On average, there were only one physician and 62 nurses available at each participating site, with an annual patient load of 36,615. Ninety-six percent of the TASSH study sites had a laboratory and pharmacy on site, although medication supply was very limited (Table 1).

**Table 1:** Characteristics of TASSH Sites

Characteristics	Total (*n* = 32)	Intervention Group (*n* = 16)	Usual Care Group (*n* = 16)	*P*-value
District Hospital (%)	16 (50%)	8 (50%)	8 (50%)	
Health Center (%)	16 (50%)	8 (50%)	8 (50%)	
Rural (%)	16 (50%)	9 (56%)	7 (44%)	
Urban (%)	16 (50%)	7 (44%)	9 (56%)	
Pharmacy available onsite (%)	31 (96.2%)	16 (100%)	15 (93.8%)	
Laboratory available onsite (%)	31 (96.2%)	16 (100%)	15 (93.8%)	
Median number of MD on staff	1.00	1.00	1.00	1.00
Median number of nurses on staff	62.00	59.50	78.00	0.72
Median number of patients per day	100.32	80.84	140.21	0.08
Median number of patients per year	36,615.00	29,508.00	51,176.00	0.08

Seventy-eight percent of the CHNs were female, with a mean age of 28.13 years (SD: 1.5), and an average healthcare work experience of 3.61 years (SD: 1.24) (Table 2). Twenty- four nurses had at least a high school diploma with 2 years of nursing training, and three nurses held a bachelors degree in nursing. The general roles and responsibilities of the nurses were to check and record vital signs (BP), register clients/patients, dress wounds, administer medication (excludes antihypertensive medications), counsel the clients, and immunize, among other duties. Except for certain duties (e.g., dress wounds, conduct welfare clinic, family planning, and daily report writing on child welfare), the CHNs’ usual responsibilities (i.e., hypertension screening) did not vary significantly from the duties they performed as part of the TASSH program. This subset of 27 nurses representing both intervention and control sites received at least five training sessions (2–3 days per session) in the WHO-PEN package over a 2-year period, as part of the parent trial. The trainings provided the nurses practical skills needed to diagnose, treat, and manage uncomplicated cases of hypertension, which would normally be under the care of physicians.

**Table 2:** Characteristics of Study Participants

Characteristic	Total (*n* = 27)
Age (mean, SD)	28.13 (1.51)
Gender (n, % Female)	21 (77.8%)
Years of nursing experience (mean, SD)	3.61 (1.24)

### Themes identified

Based on CFIR subconstructs related to implementation climate and readiness for implementation, three themes emerged from the data: patient health goal setting- relative priority and feedback from nurses; leadership engagement (in this case, medical directors); and availability of resources. The three themes identified are summarized in Table 3. The themes fit into one subconstruct of outer setting (patient needs and resources captured by patient health goal setting) and two subconstructs of inner setting (implementation climate and readiness for implementation as depicted by leadership engagement and availability of resources). The quotes provided to support the themes were taken from either the personal interviews (PI) or focus group sessions (FG).

**Table 3:** Thematic Analysis of Stakeholders’ Perceptions of TASSH Implementation Guided by Consolidated Framework for Implementation Research (CFIR)

TASSH Themes (% CHN endorsing theme)	CFIR Definition	Sample Quotes
Patient Health Goal Setting (93%)	The degree to which information about the intervention implementation is easily accessible and digestible	*“Make changes in diet, proper medication”[PI]*
		*“Cut down on salt intake, reduce intake of fatty food, exercise, eat fruits a lot, stopped smoking and drinking” [PI]*
Leadership Engagement (93%)	The commitment, involvement, and accountability of leaders and managers in implementation	*“Motivational package, separate unit for the students, support from the higher authority of Ghana health services and MOH [Ministry of Health]”[PI]*
		*“Gave me enough time to attend the TASSH training and forwarded hypertensive patients to me” [PI]*
		*“Gave us support even when our clients come and we the TASSH nurses are not around, they call us to find out what our clients should do” [FG]*
Availability of Resources (100%)	The level of resources dedicated for implementation (e.g., funds, equipment, personnel, training)	*“Availability of logistics, especially cardio check, BP apparatus; a TASSH corner or room in selected facilities for program”[FG]*
		*“BP monitoring machines, measuring tape, scale”[PI]*

CFIR Subconstructs: One subconstruct of outer setting (patient needs and resources captured by patient health goal setting) and two subconstructs of inner setting (implementation climate and readiness for implementation as depicted by leadership engagement and availability of resources)

#### Outer setting

##### Theme 1: patient health goal setting- relative priority and feedback from nurses

Overall, CHNs shared positive perceptions of TASSH and thought the program was effective in improving hypertension outcomes. Many of the nurses offered affirmative feedback for the program including, *“The study has helped enlighten patients about the risk of high blood pressure”* [FG, Nurse 4]. Also, *“It has helped many clients reduce their blood pressure”* [FG, Nurse 15]. Nurses provided their patients with motivational interviewing and behavioral / lifestyle counseling as a part of the TASSH program, which demonstrated positive effects on patient lifestyle choices and ultimately health outcomes. This was confirmed via the feedback provided to the CHNs from the patients about how the TASSH program has impacted their health. One of the nurses recounted a patient saying, “*Now I have cut down on salt intake; now I have reduced intake of fatty food; now I exercise; now I eat fruits a lot; now I have stopped smoking and drinking*” [PI, Nurse 5]. Moreover, nurses noted that their patients’ blood pressure had decreased, which may be due to adopting healthy diets and incorporating physical activity into their everyday lifestyle. For instance, as described by one nurse, “*they [Patients] are saying because of the education on lifestyle they know when to eat and when not to do something*” [PI, Nurse 13]. Furthermore, TASSH nurses felt that the program was able to encourage goal setting among the patients, based on the positive effects that the program had on blood pressure. According to one patient, *“[TASSH has] helped them to set up their own lifestyle goals*” [PI, Nurse 3]. The lifestyle goals fostered by TASSH encouraged healthy behavioral changes (medication adherence, healthy diet, and regular physical activity) in order to maintain a normal blood pressure. The constructive feedback patients received from the nurses for noticeable improvements in their health served as further motivation to continue goal setting in order to maintain a healthy lifestyle. Nurses viewed the TASSH strategy as helpful to patients in improving their condition over time, stating, for example, *“[TASSH] has helped many clients with BP to reduce it from abnormal to normal*” [PI, Nurse 9] and *“[TASSH] has helped some patients know more about their condition. It helped them come to the hospital regularly because they previously just took medication without coming*” [PI, Nurse 10].

Patients’ ability to set behavior change goals and stick to them with the guidance of TASSH nurses reflects the importance they place on health or the relative priority for improving their health outcome. A nurse discussed the attitudes and behaviors of the patients, in response to the intervention as such *“[The patients] always listen to advice and their health has improved”* [FG, Nurse 26]. The patients appreciated and made use of the information they received about hypertension and the need for lifestyle changes and adherence to medication. According to one nurse, a patient *“had a reduced BP through education on lifestyle and the importance of taking their medication”* [PI, Nurse 11]. Furthermore, there was consensus among the nurses that the program was widely accepted by most patients; a nurse stated, *“Clients react nicely about TASSH especially during follow-up visit”* [FG, Nurse 2] and *“[Patients] enter the program with kind heart and respect”* [FG, Nurse 9]. According to another nurse, “*A client said it was through TASSH that she got to know that she is hypertensive*” [PI, Nurse 8]. During the personal interviews and focus group sessions, nurses expressed that patients cared about their health and, as a result, the patients were very punctual for the hospital visits. Most patients were *“always available for follow-up”* [PI, Nurse 3] and *“they are always on time during follow-up. They were respectful*” [PI, Nurse 1]. Moreover, nurses stated that the most important reasons for patients’ participation in TASSH were as follows: motivated to improve their diet or eating habits (85%), motivated to be physically activity (62.9%), and 59.3% endorsed that patients participated to prevent diabetes or improve their health [PI, Nurses 1–27]. Nurses expressed that lack of time was a consistent barrier mentioned by patients who did not adhere to TASSH study visits, followed by clinic distance and money for transportation as well as other faith-based reasons. One nurse stated that a patient refused to participate because of spiritual belief; the patient believed that accepting she had hypertension would accelerate her death [PI, Nurse 22].

#### Inner setting

##### Theme 2: leadership engagement

According to the nurses, the support or lack of support offered by health facility leaders or medical director/supervisors also influenced the effectiveness of the TASSH protocol implementation at the various sites. The majority of the nurses interviewed indicated that their site directors were very supportive of their involvement in TASSH. Sixty-six percent of the nurses stated that their site directors were very familiar with the TASSH program. The leaders released them from other duties to allow them to focus on the TASSH program: *“We were [relieved from duties] to attend the TASSH program and TASSH visits”* [FG, Nurse 4]. Another nurse described the involvement of the leaders as follows: we *“have support for TASSH because they do think it’s an interesting program in educating nurses or training nurses to be able to care for patients with diet related diseases”* [PI, Nurse 2]. Overall, those site directors who were versed in the TASSH protocol provided their unyielding support to the nurses and ensured that *“[Nurses’] clinical priorities were in line with TASSH”* [PI, Nurse 3]. However, nurses at sites with leadership turnover expressed that *they “divide their attention and often do not have enough time to carry out their TASSH duties”* [FG, Nurse 20]. One nurse described the support provided by her site director as helpful: *“Even though I have to perform my other duties before TASSH, my site director lets me attend to TASSH patients when they come around, gives me enough time to attend TASSH training, and forwards new patients to me”* [PI, Nurse 5]. In addition to the site directors ensuring that time is allotted to the nurses for TASSH training and taking care of the patients, nurses depended on the site directors to provide the necessary space at the facility to perform TASSH duties. One nurse shared the following: *“When we requested space for the TASSH sessions, though there was no space, [the director] assured us of his support”* [PI, Nurse 17]. Furthermore, *“[The director] asked the in-charges of various departments to allow the TASSH team to use their offices”* [PI, Nurse 13].

Nevertheless, leadership support was described as critical, particularly in cases where the primary duties of the nurses at the clinic (i.e., solely maternal child duties including immunization, etc.) were not in alignment with their duties as TASSH nurses, and even more so in facilities with limited space. Based on statements provided by the nurses during the personal interviews and focus groups, majority of nurses viewed their site directors as being vested in the TASSH implementation process, committed to supporting their efforts, and assisted with resolving unexpected challenges. However, in facilities with turnover of site directors, initial leadership support was not as strong. Over time, leadership and accountability improved once the nurses made new directors aware of the TASSH program.

##### Theme 3: availability of resources

The TASSH research team provided all of the material resources and training needed to ensure efficient implementation of the TASSH protocol for the parent trial. The nurses reported the importance of having the following resources available, including: *“BP apparatus or machines, booklets for reference, on-site laboratory, health insurance, medication, weighing scale and measuring tape”* [PI and FG, Nurses 1–27]. The most helpful materials and resources identified by the nurses were BP apparatus and booklets for their reference. These contributed significantly to the successful implementation of the TASSH program. However, *“lack of space, lack of cooperation from clients, financial constraints, transportation issues, delayed laboratory results, delayed monthly salaries for the nurses, unavailability of fixed space for data collection, and loss of clients to follow-up”* [PI, Nurses 1–27] were cited as issues that may threaten the successful implementation and scale-up of the TASSH program. The nurses also suggested that the research team make available other resources to them, including glucometers, thermometers, and disease health charts. A handful also advocated for a permanent *“venue to hold TASSH program*” [FG, Nurse 11] at their facilities, eliminating the need to scramble for space to complete TASSH patient visits during follow-up. One nurse mentioned that, at times, she had to find a seat under a secluded tree to conduct her patient visit [FG, Nurse 10].

While a majority of the nurses had minimum challenge combining their regular duties with that of TASSH, two mentioned that it was difficult combining TASSH duties with their usual responsibilities. One nurse expressed, *“[I] had to divide my attention and didn’t have enough time for TASSH”* [FG, Nurse 20]. Furthermore, another explained that it is*“very busy to integrate other responsibilities with TASSH”* [FG, Nurse 16]. One of the nurses mentioned that her *“usual TASSH work at the OPD (out-patient department, where TASSH patients are seen) is far from the eye care department”* [PI, Nurse 21]*,* where she is normally stationed*.* Another had to *“go home with work to be done”* [PI, Nurse 26]. Evidently, there was a need to combine regular duties with TASSH duties at some facilities. Although some nurses mentioned they were relieved from other duties in order to satisfy TASSH obligations, about 70% of nurses stated that they received help from staff working in other departments so they could perform TASSH duties in a timely manner.

Nurses consistently noted the importance of logistics for program implementation and sustainability. The majority of nurses emphasized access to properequipment (i.e., BP machines) and training in its use. Furthermore, some nurses mentioned improving mechanisms for storing patient information and information from the TASSH training sessions. Transportation for patients and clinic workers was mentioned as a barrier to TASSH implementation and an aspect of the program that requires attention in the long term. Particularly for nurses visiting patients who reside in rural areas, there are limited “means of transportation to the villages where the patients are” [FG, Nurse 20]. Additionally, nurses whose work locations were far from their homes, expressed concerns about transportation difficulties and associated costs. Finally, space for the TASSH program was discussed extensively during the personal interviews and the focus group sessions—specifically, in many cases, the program did not have a designated space in some facilities, which may hinder future implementation efforts and sustainment of TASSH.

Overall, the nurses were generally impressed with the TASSH program, appreciative of the training they have received, and thought it was very effective in controlling hypertension. One nurse stated: *“The study has helped [patients] to reduce and enlighten them about the risk of hypertension”* [PI, Nurse 5]. Another added that *“[TASSH] helped some clients in renewing their expired NHIS [Ghana’s National Health Insurance Scheme card]”* [PI, Nurse 3] and “*It [TASSH] also helped them set up their own lifestyle goals”* [PI, Nurse 3]. However, the barriers to TASSH implementation included partial leadership support in some facilities, where nurses could only work on TASSH if and when they had time, limited material resources, and space to implement TASSH [FG and PI]. More than half of the nurses expressed that ongoing guidance from implementation science experts, including efficient implementation planning, problem solving, ensuring availability of resources, mutual respect / trust, and provision of reliable knowledge in order to carry out project tasks, are all important for successful implementation of TASSH [PI].

## Discussion

Given the burden of hypertension in Ghana, and the challenges to hypertension care and management in many LMICs [32, 33], there is an urgent need for effective strategies for hypertension control. Task shifting is a viable strategy for the reduction and control of high blood pressure in low resource settings. This study aimed to examine nurses’ perceptions of the implementation of the task shifting strategy to manage hypertension in Ghana. Using CFIR as a guide, emergent themes were grouped based on subconstructs of the following CFIR constructs: patient needs and resources, implementation climate and readiness for implementation [9]. TASSH implementation was fostered by leadership engagement, including site directors relieving nurses from clinic duties to address the needs of their TASSH patients, and to attend TASSH trainings. Site directors also provided space for TASSH consultations to occur. For individuals in leadership positions without a full understanding of the TASSH program and nurses’ duties, more in-depth and frequent education is necessary. The primary mechanism by which site leadership can facilitate the TASSH program is to allow nurses to incorporate TASSH with their typical work duties, such as providing care to TASSH patients during regular clinic hours, and if necessary, providing personnel to cover nurses’ shifts when TASSH requires their presence elsewhere (e.g., training, patient follow-ups at home).

The findings from this study reflect the implementation climate and readiness to change constructs of the CFIR model, highlighting the importance of leadership, access to resources, and education or knowledge of hypertension management. Indeed, leadership engagement facilitated TASSH implementation for many of the nurses, despite the fact that staff turnover may have affected the process in some cases. Leadership was also crucial for access to resources including space in which to conduct TASSH visits. Although the TASSH program provided blood pressure machines and materials for anthropometric measures, the nurses in the usual care group, described difficulty accessing other resources including consistent supply of medications. Additionally, lack of transportation to reach clients living in remote areas was a challenge. Some nurses described that they were unable to get to their patients for home visits especially those in the rural areas, with transportation difficulties limiting their ability to complete follow-up visits for non-compliant patients. Most of the rural areas in Ghana have underdeveloped roads with few vehicles that are able to navigate these roads. In some locations, you can only reach the village with a motor bike. Also, many health centers are quite a distance from the residences of the community members, making it difficult for rural-dwellers to access health care services. Additionally, majority of the community members in rural areas are farmers who spend substantial amount of time on the farm as their main source of livelihood. Thus, the patients would rather continue working than travel to see a health care provider, especially when they perceive health care as being unaffordable [34], in addition to trying to meet their families daily basic needs.

The feedback from the nurses brought to bear the extent to which the TASSH program has impacted the lives of the patients and the factors that influenced this change. For instance, patients’ blood pressure was reduced [19], and some patients reported positive behavioral lifestyle changes like increased participation in physical activity, and increased consumption of fruits and vegetables. The nurses attributed the modifications to the services provided by TASSH, such as the availability of BP machines, medications provided, motivational interview/lifestyle counseling, and the one-on-one patient visits, either at home or in the clinic. However, some nurses expressed that it was difficult interacting with patients who were not serious about their health, and did not see the importance of managing their blood pressure. This ultimately poses a tremendous barrier for TASSH, as part of the program effectiveness is reliant on patients’ readiness to change, and the hope that patients will incorporate the skills that they acquire into their daily lives. Although there was resistance from some patients, the majority of stakeholders appeared to have adopted the program well. Implementation of TASSH was facilitated by the perceived relevance of the intervention by both nurses and patients. For instance, the nurses described that the patients recognized the importance of changing health behaviors for improved management of their hypertension. The perceived significance of TASSH and the benefits that the program provides may foster a positive implementation climate.

Our study results concur with those of other task shifting trials for NCD management in SSA. For instance, in Cameroon, Labhardt and colleagues [17] found that shifting hypertension management duties from physicians to non-physician clinical workers was successful in reducing blood pressure. Similarly, the nurses in the TASSH program observed that over the course of the intervention, their patients’ blood pressure had improved. Another study in Nigeria that focused on task shifting from physicians to pharmacists not only found decreased blood pressure, but increased patient satisfaction [35]. Indeed, the findings from our study suggest that patients were satisfied with several aspects of the program from the nurses’ perspective. Furthermore, nurses reported that patients recognized the importance of adhereing to the program strategies for improving their health, which facilitated positive behavior changes in managing their hypertension.

Similar to our study, a previous study in Nigeria assessed opportunities, challenges and supported action for task shifting [36]. The researchers reported improved access to life-saving treatment and survival as one of the opportunities presented by task shifting. Zachariah and collegues also suggest “standardization of protocols to facilitate the decentralization of interventions to lower levels of the health system” [36]. One challenge cited was poor salaries, suggesting that low salaries have an impact on patient care. Health care workers would absent themselves from the health centers and attend workshops because of the per diem that workshop attendance would add on to their low salaries. The authors also found that a large percentage of the health workers “supplemented their income privately” [36]. The action proposed in the study was provision of decent salaries for the health workers in order to adequately cater for their daily needs [36]. Similarly, other challenges noted by the TASSH nurses included the delayed salary payments and difficulty affording the costs incurred due to travel for patient follow-ups. Furthermore, beyond monetary resources, the nurses highlighted the importance of logistics including BP equipment, space, and education, all of which are necessary for the effective sustainment of the TASSH program. This finding was supported by a cross-sectional study by Mendis and colleagues in Nigeria, which identified lack of knowledge and awareness of hypertension, as well as lack of laboratory facilities as barriers to appropriate hypertension management [33].

Overall the results underscore the importance of leadership, adequate access to resources, and education in implementing the TASSH program, and illuminate specific challenges to address as we move forward with future implementation of task shifting for hypertension management. In order for TASSH to be scaled-up and implemented in other sectors of the Ghana Health Care System, these aforementioned challenges should be addressed through continued conversations and collaboration with a variety of key stakeholders [27] including health policymakers.

A key strength of this study is that it drew upon the perspectives of nurse stakeholders who participated in a task shifting strategy for blood pressure control in Ghana, and therefore are well positioned to share their candid experience with the program. Additionally, data collected via in-depth interviews and focus group discussions allowed for triangulation, in most cases, the data from the focus groups confirmed and expanded on the information gathered from the one-on-one interviews. Nonetheless, this study is not without limitations. First, as not all the nurses were interviewed due to the purposive sampling strategy, our sample size was small. Thus, there is a possibility that the feedback we received is not representative of the views of all nurses participating in the trial. This also limits generalizability of the study findings. There is also the possibility of social desirability bias, as TASSH coordinators conducted the interviews, which may have influenced the responses provided by the nurses [37]. Furthermore, a misinterpretation in translation could have occurred as the interview questions were translated from English to Twi (the local language), and responses from Twi to English. However, this may have been minimized as multiple bi-lingual research members reviewed both versions of the transcripts.

## Conclusion

The use of CFIR theoretical framework allowed us to examine TASSH nurses’ perceptions of the TASSH program using the various constructs of inner and outer setting domains (patient needs and resources, implementation climate, and readiness for implementation). This led to uncovering three important CFIR subconstruct themes, including patient goal setting, leadership engagement (in this case medical directors); and availability of resources. Assessing stakeholders’ perception of the TASSH implementation process is crucial as it provides a platform for the nurses to evaluate the various aspects of the task shifting program, while considering the local context in which the program is implemented. The feedback from the nurses informed barriers and facilitators to implementation of TASSH within the current healthcare system, and suggested system level changes needed prior to scale-up of TASSH to other regions in Ghana with potential for long-term sustainment of the task shifting intervention. Outer setting subconstructs (i.e., patient goal setting) and inner setting subconstructs (i.e., leadership engagement and availability of resources) must be addressed prior to widespread implementation. The lessons learned from the TASSH program may inform future evidence-based task shifting strategies implemented in low resource settings in many LMICs. The findings also reinforces the importance of theory driven application to stakeholder perception of evidence-based interventions.

## Data Availability

This study is part of a larger randomized controlled trial. This paper is focused only on the perceptions and experiences of the nurses with the TASSH trial. All data generated or analyzed during this study are included in this published article. The main trial outcome paper and accompanying data is published with PLOS medicine - https://www.ncbi.nlm.nih.gov/pmc/articles/PMC5929500/

## Abbreviations

BP: Blood Pressure
CFIR: Consolidated Framework for Implementation Research
CHC: Community Health Centers
CHNs: Community Health Nurses
LMICs: Low and Middle-Income Countries
NCD: Non-Communicable Diseases
NPHCPs: Non-Physicians Healthcare Providers
RCT: Randomized Controlled Trials
SSA: Sub-Saharan Africa
TASSH: Task Shifting Strategy for Hypertension
WHO: World Health Organization
WHO-PEN: World Health Organization – Package of Essentials for Non-communicable diseases
